# Correlates of Caregiving Burden among Bedouin-Muslim Mothers of Children Diagnosed with Epilepsy

**DOI:** 10.3390/ijerph191811595

**Published:** 2022-09-14

**Authors:** Offer E. Edelstein, Talia Shorer, Zamir Shorer, Yaacov G. Bachner

**Affiliations:** 1 The Spitzer Department of Social Work, Ben-Gurion University of the Negev, Beer-Sheva 8410501, Israel; 2 Soroka Medical Center, Ben-Gurion University of the Negev, Beer-Sheva 8410501, Israel; 3 Pediatric Neurology Unit, Soroka Medical Center, Ben-Gurion University of the Negev, Beer-Sheva 8410501, Israel; 4 Faculty of Health Sciences, Department of Epidemiology, Biostatistics and Community Health Sciences, Ben-Gurion University of the Negev, Beer-Sheva 8410501, Israel

**Keywords:** epilepsy, caregiving burden, mothers, minorities, Bedouins

## Abstract

A paucity of research exists on caregiving burden (CB) and the factors associated with it among minority groups, such as Bedouin mothers of children diagnosed with epilepsy (CDE). The aim of this study was to explore associations between CB and care-recipients’ characteristics, contextual factors, and caregivers’ characteristics among those mothers. Methods: A total of 50 mothers completed self-report questionnaires while visiting pediatric neurology outpatient clinic centers, using valid and reliable measures. Results: Bivariate associations were found between social support, number of medications, and CB. General self-efficacy and place of residence emerged as significant predictors of caregiver burden. Conclusions: These findings provide health professionals with a better understanding of the factors that should be assessed in order to address caregiver burden among Bedouin mothers of CDE. Understanding the unique characteristics and culture of the Bedouin community can help professionals in targeting caregivers with a lower sense of self-efficacy, and those that reside in Bedouin cities, in order to reduce their caregiving burden.

## 1. Introduction

Epilepsy is a brain disorder characterized by a predisposition to produce epileptic seizures with neurobiological, cognitive, psychological, and social consequences [1,2,3]. Among children, epilepsy is the most common neurological disorder [4]. Estimates [5] show that the incidence of children diagnosed with epilepsy (CDE) ranges from 41–187/100,000, with a higher incidence in underdeveloped countries. Prevalence ranges from 3.2–5.5/1000 in developed countries and 3.6–44/1000 in underdeveloped countries. Epilepsy has profoundly substantial effects on quality of life [6], as well as the social and professional lives of caregivers of children with this condition [7]. Although epilepsy is a treatable condition, due to its uniqueness, parents of children with CDE must remain vigilant and responsive to their children’s evolving health situations [8], resulting in an increased caregiving burden (CB) for caregivers. Caring for CDE is associated with significant levels of CB [9,10,11,12].

Caregiving burden refers to a wide range of impacts resulting from care provision, and includes economic, behavioral, functional, social, psychological, physical, and medical domains [13,14]. The empirical literature has already demonstrated a direct link between CB and a decreased quality of life for parents of CDE [10,15]. Other studies suggest that parents’ CB also indirectly affects their quality of life through poor coping strategies [16] and feelings of inability to deal with their child’s needs [17]. CB is known to be a significant factor in explaining anxiety [9,18], depression [18,19,20], and stigma [21] in CDE parents. Nevertheless, understanding the factors contributing to the caregiving burden of parents of CDE who pertain to minority groups is far from comprehensive [22,23].

Factors associated with CB can be generally divided into three main categories: care-recipients’ characteristics, contextual factors, and caregivers’ characteristics [24,25]. Considerable research attention has focused on the characteristics of the care recipient of individuals diagnosed with epilepsy, such as seizure frequency [21,26,27], severity, unpredictability [21], and controllability [19]. The above variables play an essential role in explaining CB in parents of CDE. The central part of contextual factors—such as social support in understanding CB among caregivers of children diagnosed with chronic conditions such as cerebral palsy [28], cancer [29], and epilepsy [30]—is evidenced. Social support may also mitigate the associations between CDE and other adverse psychological outcomes such as major depression [31], increased anxiety [9], and decreased quality of life [32]. Although parents of CDE may need support from their environment, to the best of our knowledge, social support among Bedouin mothers with CDE to explain CB has not been previously studied. Finally, self-efficacy (SE), a caregiver trait, reflects the beliefs in one’s personal ability to successfully cope with unusual, uncertain, and/or stressful circumstances [33,34]. Strong SE among parents of children diagnosed with cerebral palsy is associated with lower CB [35], lower stress [36], and better psychological well-being in parents of children diagnosed with cancer [37] and pediatric type 1 diabetes [38]. Evidence suggests that high parental SE is associated with better child seizure management [39], and the performance of effective stress-related coping strategies (planning and active coping) in parents of children with intellectual and developmental disabilities [40].

In the current study, we focused on the Arab Bedouin minority. Estimates show that the Arab Bedouin population in Israel is approximately 260,000 [41]. The Bedouin are Muslim, tribal, traditional, and patriarchal [42,43], and their society holds collectivist rather than individualist values [44]. In the last decade, Bedouin society has undergone remarkable changes, such as significant urbanization processes [45], greater participation in the market and wage economy [46], and more formal education [47]. Nevertheless, compared to other minorities in Israel, including Arabs, Bedouins are the most impoverished [41] and have the highest fertility rate [41]. Half of the Bedouin in Israel live in unrecognized villages and lack basic infrastructure such as electricity, paved roads, and running water [48], and they have less access to health services [49,50].

Studies of CB among Bedouin parents of CDE might underline coping mechanisms that can be employed to alleviate CB in other minority groups in Israel and worldwide. We decided to focus on mothers for two reasons: First, mothers report more adverse care-related outcomes than fathers in various caregiving studies [51,52]. Second, in traditional patriarchal societies such as the Bedouin, women tend to fulfill the expected gender role of caregiving [44,53]. In order to develop culturally-sensitive, tailored intervention programs suited to address Bedouins’ specific needs and characteristics, an understanding of CB and its factors is essential. This research sought to explore associations between CB and care-recipients’ characteristics, contextual factors, and caregivers’ characteristics among Bedouin mothers of CDE.

## 2. Materials and Methods

### 2.1. Participants

A total of 50 Bedouin mothers of children with epilepsy participated in the study. Inclusion criteria required that participants (a) were at least 18 years of age, (b) were the primary caregivers of a child with a diagnosis of epilepsy according to currently accepted diagnostic criteria of pediatric neurologists, and (c) were able to understand and complete the questionnaires in Arabic. In order to calculate the minimum sample size for a final model with three predictors, we used Stevens’s rule [54] with a 15:1 ratio (subject to variable). We approached 61 potential mothers; five were excluded as they failed to complete the interview and six refused to participate due to emotional discomfort. The total response rate was 81.9% (50/61).

Mothers’ and children’s sociodemographic and health-related characteristics are presented in Table 1. The mothers’ mean age was about 33 years (S.D. = 5.1); the average number of years of education was about 12. All the mothers were married, with an average of 4.5 children, rated their socioeconomic status as “fair”, and reported a moderate sense of self-efficacy. The children’s mean age was 8.6 years (S.D.= 5.1); 54% were males. Most of them exhibited behavioral disturbances and were prescribed with two or fewer anti-epileptic medications. The mothers described most children as suffering from moderate or severe convulsions. The mothers reported an average of five years since the epilepsy diagnosis, and more than half resided in villages. Lastly, they reported a relatively high sense of social support.

### 2.2. Measures

#### 2.2.1. Dependent Variable

*Caregiving burden*: The abridged Zarit Burden Interview was used to measure the mothers’ sense of caregiving. This tool comprises of 12 items [55,56] consisting of two constructs—personal strain and role strain. Items were rated on a 5-point Likert scale from 0 (never) to 4 (nearly always), with a higher score representing a higher sense of burden. A total score for this scale was calculated by summing each of the item’s responses—ranging from 0 to 48. The scale was translated into Arabic and was found to be valid and reliable [57]. In the current study, internal consistency was high (α = 0.86).

#### 2.2.2. Independent Variables

*Children’s socio-demographic and health-related characteristics*: We obtained information regarding age (in years), gender (male/female), behavioral disturbances (yes/no), number of anti-epileptic medications (up to two/three and above), and severity of convulsions (mild/medium/severe).

*Mothers’ socio-demographic and care-related characteristics:* We obtained information regarding age (in years), education (in years), family status (married/other), and the number of children.

*Self-efficacy*: This construct was measured using the general self-efficacy scale [58]. This scale comprises of 10 items reflecting the mother’s personal judgment of the extent to which how well behavior can be implemented in situations that contain novel, unpredictable, or stressful elements. Responses were rated on a 4-point scale ranging from 1 (does not describe me at all) to 4 (describes me to a great extent). The total score is the average of the responses to all items; a high score reflects a strong sense of self-efficacy. The internal consistency was high (α = 0.92).

*Situational variables:* We obtained information about the duration of the illness (years) and place of residence (city/village).

*Social support:* This construct was measured using the multi-dimensional scale of perceived social support (MSPSS) [59]. This 12-item tool comprises of three primary support sources: family, friends, and significant others. Responses were rated on a 7-point Likert scale ranging from 1 (not at all) to 7 (to a great extent). An overall score was calculated by averaging the responses to all items; a higher score represents a stronger sense of perceived social support. The internal consistency was high (α = 0.92).

### 2.3. Procedure

Complying with the inclusion criteria, pediatric neurologists identified appropriate potential Arab Bedouin mothers and provided them with information about the study and its importance. Mothers interested in participating in the study were asked to sign an informed consent form and complete the self-reported questionnaires. The Helsinki Committee of Soroka University Medical Center approved the study protocol. Data obtained in this research were anonymously coded in an encrypted file and stored on the researcher’s password-protected computer.

### 2.4. Statistical Analysis

We used descriptive statistics (percentages, means, and SDs) to describe the sample and study variables. Pearson’s correlation coefficients were computed to assess the associations between CB and research variables. Differences among mean values of continuous variables were evaluated using *t*-test (Fisher’s exact test) and analysis of variance (ANOVA). Lastly, we conducted a multivariate linear regression analysis to determine the unique relative contribution of the study variables to the explanation of the mothers’ variability in caregiving burden. Only variables which correlated significantly with CB in bivariate analyses were included as independent variables in this analysis. The internal reliability of scales was assessed using Cronbach’s alpha coefficient. SPSS for Windows software (version 26.0, IBM SPSS, Armonk, NY, USA) was used for data analysis. The significance level for all analyses was set at *p* < 0.05.

## 3. Results

As can be seen in Table 1, the level of CB reported by the mothers was moderate (approx. 24 points out of 48 possible ones). Mothers’ caregiving burden was negatively associated with self-efficacy, social support, and place of residence, and positively associated with the child’s number of medications. In other words, mothers who reported lower CB tended to report higher self-efficacy and social support levels, lived in villages, and most of their children received up to two medications daily. No statistically significant associations were found between CB and children’s age, gender, behavioral disturbances, and convulsion severity. Similarly, we did not find any statistically significant associations between CB and mother’s characteristics (age, number of children, socioeconomic status) or situational variables (illness duration).

As can be seen in Table 2, self-efficacy and place of residence made a unique contribution to the mothers’ caregiving burden. Mothers who reported lower self-efficacy and those who lived in a city tended to report higher burden. The regression model was found to be significant [F (4,45) = 10.06, *p* < 0.001], and explained a high percentage (43.9%) of the variance of caregiving burden.

## 4. Discussion

This study aimed to explore associations between CB and care-recipients’ characteristics, contextual factors, and caregivers’ characteristics among one of the least investigated minority populations—Bedouin mothers of CDE. Thirty-three percent of the mothers in the current study reported that their children were prescribed three or more anti-epileptic medications. Moreover, the number of antiepileptic medications was positively correlated with CB. Our findings corroborate studies among parents of children with chronic kidney conditions that found that diminished quality of life [60] and lower levels of supportive parental behavior among parents of CDE [61] was associated with a higher number of medications. There are several possible explanations for this finding. First, although we did not find a statistically significant association between the mothers’ socio-economic status and CB, the Bedouins in Israel are the most impoverished minority population [41]. Therefore, it is reasonable to assume that certain financial constraints related to purchasing a higher number of meditations exists and may affect the overall perception of the epilepsy burden. Second, prior large-scale studies concluded that approximately one-third of the individuals diagnosed with epilepsy do not gain complete control of seizures and require more than three anti-epileptic medications [62,63]. The higher number of medications may suggest that the children’s medical situation is probably more severe and fluctuates, requiring the mothers to remain alert and respond immediately to unexpected developments [8], thus reflecting in a higher sense of CB.

The mothers in this study reported a relatively high level of social support. This finding, consistent with other studies on the Bedouin society [53,64], might reflect the core values of a collectivistic culture. The negative association found between social support and CB is comparable to findings among parents of children diagnosed with epilepsy and other chronic conditions [28,29,30]. Interestingly, social support was not a significant predictor of CB. It is worth noting that other studies among Bedouin mothers of children with developmental disabilities [53] did not find significant associations between social support and adverse psychological outcomes, such as depression and somatization. A study conducted among another collectivist society in Israel, the ultra-Orthodox Jews, showed that social support makes a relatively small contribution to explaining caregiving outcomes [65]. This might imply that despite its essential role, non-professional social support should be accompanied by other means of support to reduce the negative psychological consequences of caregiving, such as caregiver burden.

We found that self-efficacy serves as a protective attribute against caregiver burden. Previous studies among parents of children diagnosed with chronic conditions have evidenced the fundamental role of caregivers’ self-efficacy and its positive impact on stress [36], psychological well-being [37,38], and care management [39], as well as the utilization of effective, stress-related coping strategies [40]. This finding extends the understanding of the universal fundamental role of caregiver’s self-efficacy and its association with caregiving outcomes among Israel’s most impoverished minority population—the Bedouins. This finding is particularly encouraging as self-efficacy interventions have already demonstrated associations with better care-related outcomes, such as the quality of life of both individuals diagnosed with epilepsy and their caregivers [66], adequate pediatric epilepsy self-management behaviors [67], and a decrease in parental requests for unnecessary preventive interventions [68].

Lastly, we found that living in a village (compared to a city) predicated lower caregiver burden. This finding is somewhat puzzling, as living in a Bedouin village implies, in most cases, a lack of basic infrastructure such as electricity, paved roads, running water [48], and access to health services [49,50]. The Bedouin society has undergone substantial changes concerning modernization and urbanization processes [69,70]. In this instance, prior studies showed that urbanization might produce behavioral shifts from a traditional manner of living to a more modern one. Urban Bedouins are under less scrutiny of the community, with its rules and norms [71], while those who reside in villages still maintain a more strict and traditional way of life. In the current study, the lower caregiver burden expressed by mothers that reside in villages can be explained in two ways: First, it might be a reflection of greater adherence to the Bedouin society’s norms—where women naturally assume the role of caretaker [72], thus perceiving the care of CDE as less burdensome. Second, it is reasonable to assume that greater adherence to the Bedouin society’s collectivistic values and norms means a, potentially, higher level of social support. Indeed, the availability of social support is one of the virtues of the Bedouin community [73]. This might be more pronounced in less urbanized and more traditional surroundings, such as in villages. As this study sample was relatively small, it is recommended that future studies will deepen the understanding regarding the associations between place of residence and care outcomes, such as caregiving burden and related social mechanisms. Given the ongoing changes occurring in the Bedouin society, it is recommended that health professionals such as physicians, nurses, and social workers should regularly evaluate CB among these mothers and provide them with adequate information on ways to cope with the evolving challenges of epilepsy. The development of culturally-sensitive, tailored intervention programs suited to address Bedouins’ specific needs and characteristics are also warranted.

Limitations: Several limitations of our study should be noted. First, we used a cross-sectional design with a relatively small sample size, and no causal associations are assumed. Second, we recruited the participants from a single region of the country; thus, the sample may not include a complete representation of the Bedouin population, and the results are not generalizable to other regions. Third, we did not assess the assistance that the mothers may have received from governmental social services, which might explain some differences in CB. Fourth, we did not evaluate the existence of other individuals in the family diagnosed with chronic conditions, which might be associated with the CB reported in the current study.

## 5. Conclusions

Despite these limitations, our study provides health professionals with a wider understanding of the factors that should be assessed in order to address caregiver burden among Bedouin mothers of CDE. We demonstrated the fundamental roles of self-efficacy and place of residence as protective factors for decreasing parental care-related burden. Based on our findings, designated psychosocial interventions may be developed for Bedouin mothers of CDE to address their CB. These programs need to focus on strengthening mothers’ self-efficacy, as previous studies have demonstrated its important role in various care outcomes. Given the pivotal role of the CB in explaining other care-related outcomes, it is recommended that CB will be regularly evaluated among the caregivers’ population. Particular attention should be given to mothers who reside in cities, as they might be prone to higher CB, which might be associated with other deleterious, care-related outcomes such as diminished quality of life and elevated anxiety and depression.

## Figures and Tables

**Table 1:** Mothers’/children’s characteristics and associations between study measures and mothers’ caregiving burden.

Variable	No. of Items	Range	*n* (%)	M (S.D.)	Association with Caregiving Burden
Burden of care	12	0–48		24.40 (10.81)	----------------
*Child’s characteristics*					
Age	1	1–21		8.61 (5.08)	*r* = 0.03
Gender	1				
Male			27 (54.0)	23.85 (11.41)	*t* = −0.23
Female			23 (46.0)	24.59 (10.68)	
Behavioral disturbances	1	1–2			
Yes			32 (64.0)	25.96 (9.74)	*t* = −1.04
No			18 (36.0)	22.44 (13.04)	
Number of anti-epileptic medications	1				
Up to two			33 (66.0)	21.33 (11.04)	*t* = 2.87 **
Three and above			17 (33.0)	30.47 (8.03)	
Severity of convulsions	1	1–3			
Mild			14 (28.0)	19.01 (11.24)	*F* = 2.19
Medium			21 (42.0)	26.05 (11.39)	
Severe			15 (30.0)	27.07 (9.07)	
*Mother’s characteristics*					
Age	1	23–48		33.08 (5.16)	*r* = 0.01
Years of education	1	0–24		12.20 (3.95)	*r* = 0.03
Number of children	1	1–10		4.49 (2.26)	*r* = 0.14
Socio-economic status	1	1–5		2.94 (3.01)	*r* = −0.015
Self-efficacy	10	1–5		3.51 (1.03)	*r* = −0.60 **
*Situational variables*					
Duration of illness (years)	1	0.5–19		4.93 (4.58)	*r* = −0.16
Place of residence	1	1–2			
City			22 (44.0)	27.95 (8.84)	*t* = −2.14 *
Village			28 (56.0)	21.61 (11.51)	
Social support	12	17–84		53.50 (19.60)	*r* = −0.43 **

* *p* < 0.05, ** *p* < 0.01.

**Table 2:** Predictors of mothers’ caregiving burden: Results of multivariate linear regression analysis.

Variable Measured	B	S.E	β	T
Self-efficacy	−5.41	1.24	−0.51	−4.36 **
Social support	−0.11	0.17	−0.71	−0.63
Place of residence ^	−5.65	2.34	0.26	−2.41 *
Child’s number of medications #	4.46	2.73	0.19	1.63

Adj R^2^ = 0.439 ^ 1 = city, 2 = village; # 1= up two medications, # 2 = three and above medications. * *p* < 0.05, ** *p* < 0.01.

## Data Availability

Parts of data presented in this study are available on request from the corresponding author.
